# Promoting Physical Activity Among University Students During the COVID-19 Pandemic: Protocol for a Randomized Controlled Trial

**DOI:** 10.2196/36429

**Published:** 2022-06-13

**Authors:** Aurelie Goncalves, Caroline Bernal, Karim Korchi, Maxence Nogrette, Maxime Deshayes, Antony G Philippe, Béatrice Gisclard, Elodie Charbonnier

**Affiliations:** 1 APSY-V Université de Nîmes Nîmes France; 2 Projekt Université de Nîmes Nîmes France

**Keywords:** physical activity, psychological factors, university student, COVID-19

## Abstract

**Background:**

Since the beginning of the COVID-19 pandemic, sanitary context and e-learning have greatly modified student lifestyles and led to deteriorations in their mental health. An increase in anxiety and depressive symptoms and sedentary behaviors, reduction in physical activity, and a stronger tendency to move toward unhealthy diet have been demonstrated. This finding highlights the need for innovative interventions to promote healthy lifestyle among students.

**Objective:**

This research protocol aims to evaluate the effects of an intervention program on the lifestyle and psychological state of students.

**Methods:**

Students from University of Nîmes were recruited and randomly assigned to 1 of 2 following conditions: an intervention group and a control group. Participants in the intervention group were engaged in an 8-week physical activity program. Prior to the start of the program, design-based innovative workshops were conducted with participants to ensure that the program was co-constructed by the users and met their specific needs. Students in the control group did not receive any intervention. For each group, measures of physical activity, sedentary time, anthropometric data, sleep, physical condition, and psychological variables (eg, anxiety, depression, motivation, body appreciation, perceived control, well-being) were conducted at baseline and 9 weeks later.

**Results:**

A total of 110 participants were initially included. Reporting of the results is projected for the spring of 2022.

**Conclusions:**

It is anticipated that this innovative intervention co-constructed by pairs will promote a healthier lifestyle and psychological health in students. There is every reason to believe that a mobilized co-construction approach is a promising strategy to limit unhealthy habits and promote physical activity while increasing motivation. The development and evaluation of interventions to address the specific needs of university students is essential and could be transferred to other vulnerable populations such as people with chronic diseases or older people.

**Trial Registration:**

ClinicalTrials.gov NCT05019482; https://clinicaltrials.gov/ct2/show/NCT05019482

**International Registered Report Identifier (IRRID):**

DERR1-10.2196/36429

## Introduction

### Background

The COVID-19 pandemic is one of the most profound crises of our time. The socioeconomic impact of the crisis has been devastating, as have the repercussions on the well-being of the populations. In France, the general population has undergone 3 confinements (March 17 to May 10, 2020; October 30 to December 15, 2020; and April 3 to May 3, 2021). Since the beginning of the COVID-19 pandemic, university students have not been spared and have faced many challenges. They have had to adapt to many constraints (eg, reduced number of students in classrooms, wearing masks) and significant changes in teaching (eg, distance and/or hybrid education). It was only at the beginning of the academic year September 2021 that the university has returned to an almost normal functioning (ie, almost all face-to-face teaching with the full complement of students but with the wearing of masks maintained). However, the re-increase in COVID-19–positive cases since November 2021 suggests that students’ conditions could be affected again in the coming weeks.

Students are among the population for whom the pandemic has had the most negative impact on their lives [1]. Even before the pandemic, university students were identified as a population with unhealthy lifestyles and habits, notably reflecting unhealthy diet [2], poorer mental health than their nonstudent peers [3], high levels of sedentary behavior (SB), and low levels of physical activity (PA) [4,5]. During the pandemic, and more specifically during the first lockdown, this unprecedented sanitary crisis clearly had a massive impact on the lifestyle and mental health of university students around the world [6], and French students have not been spared [7,8]. Studies conducted during the first lockdown have shown an increase in unhealthy lifestyles as evidenced by their high levels of SB [9,10] and their low levels of PA [8,11,12]. In the same vein, high levels of anxiety and depressive symptoms among university students were reported during the first lockdown in different countries around the world [13-15], just like in France [7,16]. The few longitudinal studies conducted during the following lockdown attest to a maintenance of the difficulties or even a worsening of them [7,17-19]. The authors suggest that adverse health effects will persist long after the COVID-19 pandemic and note the need for long-term interventions [20,21]. In this perspective, interventions in PA appear particularly appropriate. Indeed, even if this was already known before, this pandemic reinforces the protective role of PA on physical and mental health [22,23]. This has led many governments and sports science societies to provided recommendations on PA and SB during the COVID-19 pandemic, particularly to prevent the occurrence or worsening of chronic diseases [24-26]. Despite these recommendations, very few interventional studies to promote PA and reduce SB have been conducted.

As noted above, students were particularly affected by the COVID-19 pandemic and were already a vulnerable population before the pandemic. Therefore, they are a prime target for this type of intervention. It is essential to conduct interventional studies with university students to accompany them in this (even more) complex period and prevent the deterioration of student health in a COVID-19 context. However, getting students to engage in health promotion interventions is often difficult. Indeed, many students do not seek help [27] due to barriers such as stigma or lack of information [28]. Therefore, it is essential to go beyond traditional interventions and provide more innovative and accessible interventions to achieve real student engagement. This is especially important because if we don’t implement interventions to which students adhere, we may see lasting alterations in their lifestyles and, more broadly, in their health.

### Study and Protocol Aim

This study is conducted in order to reduce or prevent deterioration of the health of university students related to the COVID-19 pandemic. Specific objectives of this study are to measure the impact of this innovative program on physical capacities, lifestyle, and psychological issues. We hypothesized that our program can have beneficial effects on university students’ physical capacities, improve their motivation to engage in PA, reduce their SB, and improve psychological issues.

To this end, students from the University of Nîmes have benefited from an innovative intervention aimed to improve their motivation to engage in PA and reduce sedentary lifestyle. First, co-construction workshops were conducted with participants to define the modalities of the expected PA program. Once the program was co-constructed between pairs, they participated in the new program. This paper aims to describe the protocol of this study.

## Methods

### Context

The COVID-19 pandemic has increased unhealthy lifestyles of students that were already prevalent before the pandemic. Their difficulty in engaging in PA programs justifies the need for innovative interventions to address their specific needs. First, in February 2021, our team conducted a pre-study to evaluate the effects of a PA program conducted by students for other students to promote PA and social interactions. However, the restricted access of students in the university to minimize COVID-19 propagation did not permit us to obtain a large sample size. In order to conduct a larger study, we have developed a research project called Cov’Etu. It was submitted in April 2021 in the framework of a call for proposals about COVID-19 (AAP Résilience–COVID-19) managed by the French research agency (Agence Nationale de la Recherche) and approval was granted. The Cov’Etu project includes 2 axes of research. The first axis aims to identify the role of individual factors (eg, symptoms of COVID-19, health concerns) on students’ psychological health (eg, anxiety and well-being) and lifestyle (eg, SB, alcohol) over the course of the pandemic. The second axis of research evaluates the health effects of 2 programs. Our research protocol refers to one of these programs as it is called in French, UNIMES EN FORME.

### Population and Study Design

This study was conducted among students from the University of Nîmes. Nîmes is a midsize city in the southern part of France that has 4 university campuses. In academic year 2021/2022, 5201 students were registered at this university, of which approximately 4000 are on the main university campus named Vauban. This campus site is located in the heart of the city and includes the administrative and sports infrastructures in addition to auditoriums and classrooms. The inclusion criteria were as follows: (1) have their courses mainly on the Vauban campus (this campus site includes students in the bachelor and master levels mainly in psychology, law, and art/design); (2) be aged 18 years or older; (3) have never benefited from any intervention in the field of PA; and (4) sign a consent form to participate in the study. Three principal exclusion criteria were established: (1) with physical diseases that prevent PA practice (eg, cardiovascular disease), (2) in sport sciences, and (3) not signing the consent form to participate in the study.

**Figure 1:**
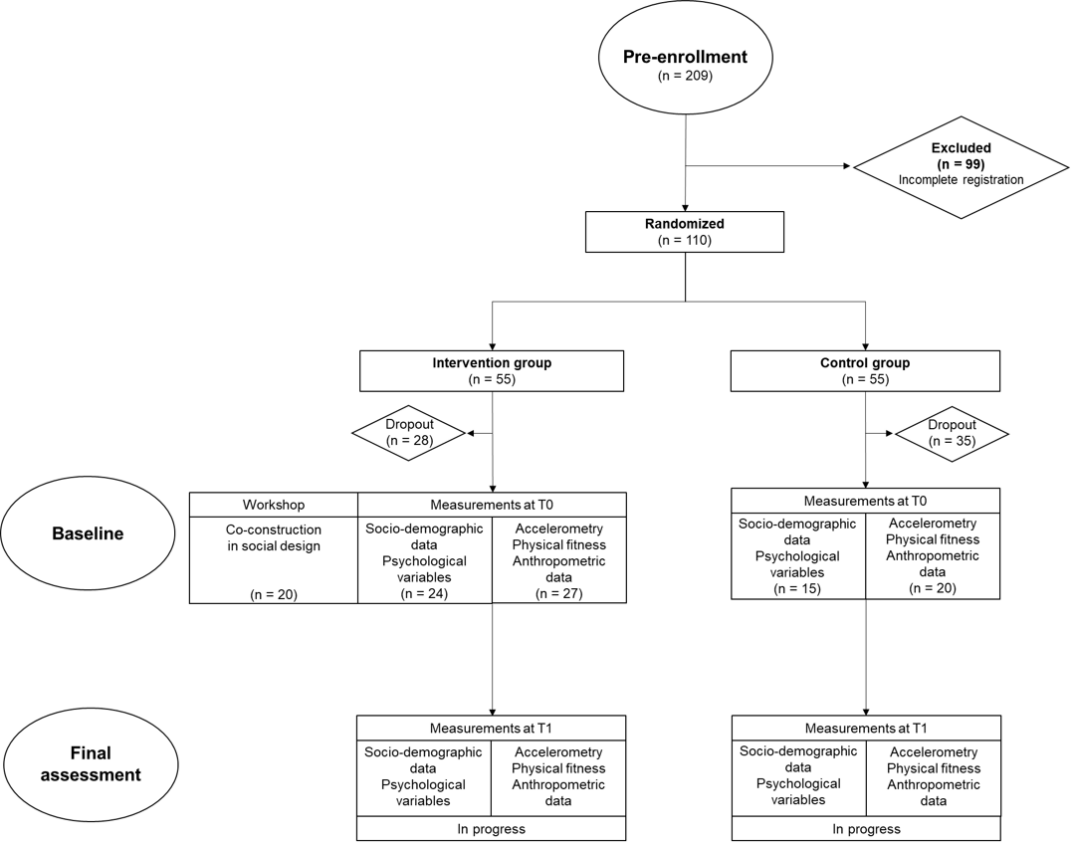
Flowchart of the study.

The study was designed as a randomized controlled trial with 2 groups. The first was the intervention group including participants who have benefited from a co-construction workshop and an innovative PA program based on their workshop. The second was the control group, which included students who had not benefited from any intervention. In total, 110 university students agreed to participate in the program, with 55 students in the intervention group and 55 in control group and randomization by age and gender. The number of students enrolled and chronological stages of the study are reported in the flowchart (Figure 1).

### Ethics Approval

An ethics review was not applicable for this study because all personal data was collected anonymously and the program evaluated has been integrated into the University Service of Physical and Sports Activities at the Université de Nîmes (governed by decree No. 2018-792 of September 13, 2018 relating to university common services) established by the Ministry of Higher Education, Research and Innovation.

The recruitment of the students was done with the approval of the Université de Nîmes health service to ensure good physical condition of the participants and was available to students throughout the program. Participants agreed to participate in this study after reading a consent form. They were informed that their personal data would remain anonymous, their participation was voluntary, and they could withdraw at any time. The study was conducted in accordance with the Declaration of Helsinki.

### Procedure

First, a workshop with the participants of the intervention group was realized to co-construct the PA program in order to remove barriers to the practice of PA. The aim of co-construction with users was to obtain better adherence to the program by being based on needs of the users themselves and not imposed by others. Second, the co-constructed PA program was conducted with them for 8 weeks with sessions twice per week.

For both groups, during September/October 2021, baseline assessments (T0) were conducted: measurements of PA and sedentary time (ST; with an accelerometer), physical fitness and body composition (face-to-face measurement), and psychological variables (online assessment). The final assessments (T1) were conducted in December 2021.

### Variables Measured

#### Physical Activity and Sedentary Time

The levels of PA and ST were measured in baseline and final assessments with the tri-axis accelerometers in the GT3X activity monitor (ActiGraph LLC) as a valid objective measure [29,30]. Participants wore the accelerometer on the right side of the hip, adjusted with an elastic belt, during 7 consecutive whole days including day and night. They removed it only during the shower and aquatic activities. ActiLife Lite Pro software (version 6.13.4, ActiGraph LLC) was used to extract PA and ST values. Data were downloaded in 10-second epoch to measure PA and ST.

Data collection was performed over 5 weekdays and 2 weekend days, and the first day of measure was excluded from the analysis. Corresponding to the French cultural schedule, PA and ST were analyzed for the whole day (6:00 AM to 11:59 PM) for weekdays and weekend days. Non–wear time during this period was excluded from the analysis, and the Choi algorithm was used to define non–wear time during the whole day as it is more adapted for the adult population [31]. This algorithm identified as non–wear time an activity of 0 counts over a period of at least 90 minutes, allowing a quota of time 1 to 2 minutes of no activity counts detected both in the 30 minutes before and after this interval. A 10-second epoch was then used to detect more accurately the changes in PA intensities [32,33]. To establish the different intensity categories (with step count and time spent in each category), the Freedson algorithm was used [34]: ST 0-99 counts per minute, light PA 100-1951 counts per minute, moderate PA 1952-5724 counts per minute, vigorous PA 5725-9498 counts per minute, very vigorous 9499 or more counts per minute.

According to the optimal methodological approach for accelerometry, 2 conditions had to be met concerning the minimum wear time required to be considered as a valid measure: (1) participants had to wear the accelerometer at least 80% of the time between 6:00 AM and 11:59 PM and (2) 70% of participants had to wear the monitor during this period [29]. To estimate usual PA and ST levels, wear-time had to be sufficient for a least 3 weekdays and 1 weekend to be considered valid [30]. All the data were processed with the ActiLife software. We focused on 7 variables for the whole day in weekdays and weekend days: number of sedentary breaks occurring in the day, ST (minutes), light PA (minutes), moderate PA (minutes), moderate to vigorous PA (minutes), vigorous PA (minutes), very vigorous PA (minutes), and number of steps.

#### Sleep

The ActiGraph GT3X activity monitor was used to measure sleep patterns. These measurements were conducted over the same period of time as the PA and ST. Participants were asked to wear the accelerometer on the right side of the hip even during the night. Sleep data were validated and analyzed if the total sleep period time was above 160 minutes per night with an estimated 90% wear time [35,36]. Data were analyzed only for participants having 3 nights validated on weekdays and 1 night validated on weekend days [35]. Raw data were downloaded in 10-second epochs using the ActiLife software and reintegrated to 60 seconds to perform sleep pattern analysis. We then applied the Tudor-Locke algorithm developed automatically by the ActiLife software and validated [37]. In contrast to previous algorithms, The Tudor-Locke algorithm provides a more accurate estimation of sleep duration and captures total sleep time from sleep onset to sleep offset, including the number and time of awakening. We focused on 7 variables for weekdays and weekend nights: total sleep time (time scored as sleep during the sleep period of the night, expressed in minutes), wake after sleep onset (time scored as wake occurring after sleep onset and before sleep offset, expressed in minutes), and sleep efficiency (total sleep time divided by sleep period time, expressed in percentages).

#### Physical Fitness

Participants in the control and intervention groups performed tests for physical fitness at preintervention (T0) and postintervention (T1). During 15 consecutive days, 180 sessions of 1-hour duration from 9:00 AM to 6:00 PM were dedicated to these measurements. The first 8 days were allocated to test the participants in the intervention group, and the last 7 days were reserved for the participants in the control group. At T1 (ie, after the intervention program), this organization was switched in order to have 8 weeks between the first and second assessments for the participants in each group. Assessments were conducted individually in a quiet university classroom. Two research engineers conducted the measurements in absolute confidentiality. Upon arrival, participant blood pressure, heart rate, and body composition were measured. Physical fitness tests were performed in order to measure balance, flexibility, lower limb strength, and cardiovascular fitness. The organization and rank of the tests was similar for the 2 groups of participants.

#### Balance

In the unipedal stance test, participants stood barefoot and held the position in a single-legged balance for 1 minute [38]. The time spent in the unipedal position was recorded in seconds. If the participant did not achieve the full minute in the balance position, the precise time spent in this posture was reported; if participant achieved it completely, the maximum time of 60 seconds was reported.

#### Flexibility

Participants completed a traditional sit-and-reach test to measure lower back and hamstring flexibility [39]. In a seated position with legs extended, participants reached as far as possible along a measured line with hands on top of each other or side by side. Measurement was reported 3 times with performance recorded in centimeters.

#### Lower Limb Strength

For assessment of muscular strength, participants were seated on a dynamometric chair (LegControl V2.0, Mtraining) with a 90° hip angle, 90° knee angle, and straps fixing the hip and thighs. The axis of the dynamometer was aligned with the anatomical knee axis. A lever, connected to the calibrated load cell, was positioned against the right leg 5 cm proximally to the medial malleolus. After a familiarization consisting of 5 submaximal isometric contractions, participants performed 3 maximal voluntary contractions of 3 seconds, separated by 1 minute each. Specifically, they were asked to contract the knee extensors as hard as they could for 3 seconds. Participants were given strong vocal encouragement during each maximal voluntary contraction. The maximum value was used for statistical analyses.

#### Cardiovascular Fitness

Participants completed a YMCA 3-minute step test [40]. Participants stepped up and down 24 steps per minute without stopping and with a frequency indicated by a metronome set to 96 beats per minute. Once the test completed, participants sat down immediately. After 5 seconds, the recovery heart rate was monitored 5 seconds during 1 minute and this last heart rate was reported to assess cardiovascular fitness [40,41]. The score was then reported on age-adjusted standards based on guidelines for gender, in 1 of 7 categories (very poor, poor, below average, average, above average, good, excellent).

#### Body Composition

Baseline standing height (cm) was recorded to the nearest 0.1 cm using a portable stadiometer (Leicester HR001, Tanita). Body weight (kg) was measured using a calibrated scale (780 MA-S, Tanita) to the nearest 0.1 kg. BMI (kg/m^2^) was calculated using height and body weight measurements. Variables were assessed through a bioelectrical impedance analysis method using a 780 MA-S (Tanita) body composition analyzer/scale such as body fat, body muscle, body water expressed in mass (kg) and percentage (%), and visceral fat rating expressed on a scale from 1 to 60. A score from 1 to 12 is considered healthy and a score from 13 to 60 excessive.

#### Questionnaire

Measurements were made before and after the intervention using an online questionnaire via an online survey designed with Qualtrics software (Qualtrics). The questionnaire can be found in Multimedia Appendix 1.

#### Sociodemographic and Situational Factors

Sociodemographic factors such as age, gender, level of education, and field and year of study were collected. In addition, situational factors on a Likert scale (0 to 100) were collected such as the extent to which participants felt that lockdown was compromising their future job prospects; the extent to which university studies were essential to participants; participants’ level of concern about their relatives’ health due to the COVID-19 crisis; participants’ level of concern about their health due to the COVID-19 crisis; and others on dichotomous answer (yes or no) such as the presence or absence of COVID-19 symptoms and the presence or absence of COVID-19 symptoms in their relatives.

#### Subjective PA

The validated French version of the International Physical Activity Questionnaire–Short Form was used to assess participant PA over the last 7 days. Seven items of the questionnaire evaluated the number of days participants performed moderate-intensive PA, walking activities, and the time (hours and minutes) spent per day in performing the exercises at those intensities. The variable was the total PA expressed as metabolic equivalents of task in minutes per week (METs/week), which is calculated as the sum of 3 PAs such as walking and moderate-intensive PA. For the categorical division into the 3 activity levels (low, moderate, and high), definitions from published evaluation guidelines were used.

#### Motivation for PA

To assess participant motivations for PA, the French version of the Echelle de motivation envers l’activité physique en contexte de santé [42] was used. This questionnaire contains six 3-item subscales assessing intrinsic motivation, integrated regulation, identified regulation, introjected regulation, external regulation, and amotivation. Participants responded on a 7-point Likert scale (1=strongly disagree to 7= strongly agree).

#### Subjective Sleep Quality

Sleep quality was assessed with the French adaptation of the Pittsburgh Sleep Quality Index [43]. Participants indicated their usual bedtime, how long it took them to fall asleep, their usual waking time, and their usual hours of sleep per night, as well as responding to different questions on a 4-point Likert scale (0=not during the past month to 3=three or more times a week). Algorithms were used to generate 7 component scores and a global total score (for scoring algorithms, see Buysee [44]).

#### Body Image

Assessment of body image was measured with the Body Appreciation Scale–2 [45]. The scale comprises 10 items rated on a 5-point Likert scale (1=never to 5=always), with higher total scoring indicating more positive body appreciation.

#### Eating Behaviors

Assessment of eating behavior was measured with the French version of the Eating Attitudes Test [46]. This questionnaire is a self-administered questionnaire that reveals abnormal eating behaviors. It consists of 26 items with 6 components scored on a 4-point Likert scale (0=never to 3=always). The total score ranges from 0 to 78, and a score ≥20 is considered to represent abnormal eating attitudes or behaviors.

#### Anxiety and Depressive Symptoms

Assessment of anxiety and depressive symptoms was measured with the French version of the Hospital Anxiety and Depression Scale [47]. This 14-item self-report questionnaire assesses anxiety symptoms and depressive symptoms (7 items for each dimension) with labels varying from one item to the next. Scores range from 0 to 21 for each dimension, with higher scores reflecting higher levels of anxiety or depressive symptoms.

#### Social Support

Assessment of social support was measured with the French validation of the Social Provisions Scale–10 item [48]. The 10 items are rated on a 4-point Likert scale (1=strongly disagree to 4=strongly in agreement). This self-report scale captures 5 dimensions of social support (2 items per dimension of support): emotional support or attachment, social integration, reassurance of worth, tangible help, and orientation.

#### Well-being

Assessment of well-being was evaluated with the French validation of the Psychological Well-being Scale [49]. The 18 items are rated on a 6-point Likert scale (1=disagreement to 6=agreement). This self-report scale captures 6 components of well-being (3 items per component): autonomy, control of the environment, personal development, positive relationships, giving meaning to life, and self-acceptance.

#### Perceived Fatigue

Assessment of perceived fatigue was evaluated with the French validation of the Multidimensional Fatigue Inventory–20 item [50], a brief self-report instrument that assesses 5 dimensions of fatigue: general fatigue, physical fatigue, mental fatigue, reduced activity, and reduced motivation. Respondents indicate their level of agreement with fatigue-related statements on a 5-point Likert scale (1=yes, that is true to 5=no, that is not true). Possible scores for each dimension range from 4 (no fatigue) to 20 (maximum fatigue). The primary outcome measure for this study will be the total of 4 subscales (general fatigue, physical fatigue, reduced activity, and reduced motivation).

#### Perceived Control

Assessment of perceived control was evaluated with the French validation of the Pearlin Self-Mastery Scale–Perceived Control [51]. This questionnaire consists of 7 items designed to assess 1 aspect of psychological coping resources on a 7-point Likert scale (1=strongly disagree to 7=strongly agree). Higher scores correspond to higher mastery.

### Intervention Program

During the first week, participants from the intervention group were invited to a meeting to co-create a PA program. Two workshops (10 participants per group) were organized in order to clarify the students’ expectations. The objectives of these workshops were as follows: (1) understand their motivations to engage in PA and/or sports, (2) understand the barriers and levers to these engagements, (3) determine the types of sports and/or PA that particularly interest both athletic and nonathletic people, and (4) determine their understanding and perception of communication supports on these topics.

The PA program was established according to these workshops. It consisted of weekly sessions (on the day of the participant’s choice) less than an hour in length. Activities such as cross-training and cardioboxing make up most of the program sessions. Table 1 provides an example of cross-training and cardioboxing sessions. Cross-training consisted of exercises organized in circuit training with kettlebells, swissballs, slamballs, battle rope, suspension straps, and elastic bands. Cardioboxing consisted of exercises such as shadow boxing, kickboxing, and muay Thaï sequences organized in several rounds. Once a week, a team sports event was organized (volleyball, baseball, tchoukball, spikeball, etc). The PA program was designed to develop strength, endurance, and flexibility/mobility in each session. Participants participated in at least 1 session a week and had a privileged access to all on-campus sports activities without registration. To communicate with participants about the program, the schedule, or any other important information, a group was created on the Discord social media platform with a subgroup for each main topic.

**Table 1:** Examples of the main physical activities.

Parts of the training session	Cardioboxing	Cross-training
Warm-up	A progressive warm-up was proposed with boxing moves, athletic moves, and running in order to prepare participants for the sequences of the session.	A progressive warm-up was proposed in circuit on each exercise without any weights and then with weights, fitting the level and skills of each participant.
First part	Participants were in pairs and started either with gloves or punching pads. They then changed places. They performed the sequences with a fixed work time and rest time.	Participants were invited to choose an exercise to start the circuit. They then performed each exercise with a fixed work time and rest time.
Second part	A new sequence and a new fixed work time and rest time was proposed, with either the work time increasing or the rest time decreasing.	Jumps, slamball shots, or mainly cardio exercises were proposed as a challenge in the remaining time. The main goal was to achieve the highest number of repetitions possible.
Mobility and flexibility	Participants were invited to either use foam rollers combined with mobility and flexibility exercises or to relax with Jacobson progressive muscle relaxation.	Participants were invited to either use foam rollers combined with mobility and flexibility exercises or to relax with Jacobson progressive muscle relaxation.

### Statistics

To further analyze changes in anthropometric values, physical fitness, objective and subjective PA and ST, sleep, and psychological variables, statistical comparisons might include (but will not be limited to) *t* tests, analysis of variance, Mann-Whitney *U* test, Kruskal-Wallis test, Pearson/Spearman correlation analyses, or linear and multiple regression analyses. Analyses will be performed using JASP software (version 0.14.1, JASP Team).

## Results

For this trial, it was hypothesized that the intervention program would improve physical capacities, increase engagement in PA, decrease SB, and improve psychological issues among the intervention group versus the control group. A total of 110 participants initially agreed to participate in the study, with a distribution of 55 participants per group. At baseline, in the intervention group (mean age 22.5 [SD 3.2] years), only 49% (27/55) of participants completed the fitness tests and only 44% (24/55) completed the questionnaire via the internet. In the control group (mean age 21.8 [SD 6.6] years), fitness test and questionnaire completion rates were lower, at 36% (20/55) and 27% (15/55) participants, respectively. Table 2 outlines key aspects of participant characteristics.

**Table 2:** Characteristics of survey respondents (n=51).

Characteristics			Value, n (%)
**Sex**			
	Female		37 (73)
	Male		14 (27)
	Other		0 (0)
**Level of education**			
	**Undergraduate**		35 (69)
		First year	7 (14)
		Second year	15 (29)
		Third year	13 (26)
	**Master’s**		15 (29)
		Fourth year	12 (24)
		Fifth year	3 (6)
	PhD		0 (0)
	Undefined		1 (2)
**Subject**			
	Psychology		24 (47)
	Languages		2 (4)
	History/geography		3 (6)
	Sciences		3 (6)
	Art/design		8 (16)
	Law/economics/management		6 (12)
	French literature		1 (2)
	Mathematics		2 (4)
	Others		2 (4)
**Positive COVID-19 test or symptoms**			
	Yes		4 (8)
	No		35 (69)
	Undefined		12 (24)
**Relative with positive COVID-19 test or symptoms**			
	Yes		21 (41)
	No		18 (35)
	Undefined		12 (24)

## Discussion

### Expected Results

This protocol paper describes the methodology processes of an innovative intervention, encompassing both PA and psychosocial components, designed to help university students. We expect that our intervention based on a co-construction program will lead to an improvement in students’ motivation to engage in PA, reduce their SB, and have positive effects on their psychological states, which appears to be even more essential during and after COVID-19 pandemic.

Indeed, the COVID-19 pandemic has profoundly influenced our behaviors in terms of PA and SB at all ages of life [28], especially among university students [52]. This study is in line with a recent systematic review on PA and physical capacities among students [53] that revealed it is crucial to get their awareness and have them maintain a satisfactory level of PA and physical fitness.

Moreover, unlike most studies that conduct conventional PA programs without taking into consideration the expectations of the participants [54], our study includes an innovative protocol based on users’ preferences. In this study, the co-construction of a PA program was used as a creative way to re-engage students in PA and modify their lifestyles, which have been deteriorated during the COVID-19 pandemic.

This approach seems particularly interesting because it has been shown that a co-constructed participative workshop was efficient to promote healthy eating habits in a socioeconomically disadvantaged population [55] and for children [56,57]. Engaging in a chosen and desired recreational PA could maintain active behaviors through healthy lifestyle habits [58].

Finally, while most studies mobilize either objective or subjective measures, our protocol proposes to cross the two in order to provide as many insights as possible on the effects of our program. For illustration, our research protocol includes several objectives measures (eg, anthropometric values, flexibility, strength) and subjective measures (eg, psychological variables) but also double measures (accelerometry and questionnaires) for PA and SB as well as sleep in order to compare the measures with this population.

### Limitations

Despite the inclusion of 110 participants, the loss of participants was substantial even before the program began. This can be partly explained by the lack of time at the beginning of the academic year or the fear of doing a PA with others during a pandemic period. It may also be due to the requirement to complete different assessment sessions (physical fitness and questionnaires, at approximately 30 minutes each) and 5 days of accelerometer wearing for baseline and T1 measurements. However, it is important to note a high adherence of participants to the PA program, suggesting that the engagement of the students who remained in the program was high.

### Conclusion

To the best of our knowledge, there have been no comparable protocols conducted on students, even more so during COVID-19. The pandemic has highlighted the need to pay more attention to the physical and psychological health of students. For this, the development and evaluation of innovative interventions to address their specific needs is essential and could be transferred to people with chronic diseases or older people. In the future, this co-construction approach could be used later by organizations and universities in order to limit unhealthy habits and promote PA while increasing motivation.

## Appendix

Multimedia Appendix 1 Questionnaire.

Multimedia Appendix 2 Peer-review report from the Agence Nationale de la Recherche (ANR).

## Abbreviations

PA: physical activity
SB: sedentary behavior
ST: sedentary time
