# Supplementary material for: Promoting Physical Activity Among University Students During the COVID-19 Pandemic: Protocol for a Randomized Controlled Trial
Source: JMIR Res Protoc. 2022 Jun 13;11(6):e36429. doi: 10.2196/36429 (PMC9202516; doi:10.2196/36429)
Supplement: Multimedia Appendix 1 [file resprot_v11i6e36429_app1.pdf]

Questionnaire – French version

Bonjour,
Nous vous proposons de participer à une recherche dont le but est d'évaluer l'état de santé des étudiant.e.s (p ex., sédentarité, activités physiques, ...) sur deux temps différents. L'objectif principal de notre recherche est de déterminer les effets d'un programme d'activité physique de 8 semaines sur les étudiant.e.s.

Ainsi, si vous acceptez de participer à la totalité de cette étude, nous vous solliciterons de nouveau courant décembre, par mail, pour répondre à un questionnaire en ligne. Il est important d'aller jusqu'à la fin du questionnaire.

Pour vous remercier de votre engagement, des étudiants seront tirés au sort et recevront des cadeaux.

L'investigateur principal de cette étude est Aurélie GONCALVES (Maître de conférences à l'université de Nimes), que vous pouvez contacter à l'adresse suivante : aurelie.goncalves@unimes.fr. Le promoteur de cette étude est l'Université de Nîmes, 5 rue du Docteur George Salan, 30000 Nîmes. Cette étude bénéficie d'un financement de l'Agence Nationale de la Recherche (ANR).
Engagement des participant.e.s : la participation à l'étude consiste à répondre à un ensemble de questionnaires, durant environ 25 minutes, et à accepter d'être recontacté pour y répondre à nouveau en décembre Les participant.e.s s'engagent à répondre le plus sincèrement possible aux questions. *Engagement de l'investigatrice :* Je m'engage à mener cette recherche selon les dispositions éthiques et déontologiques, à protéger l'intégrité physique, psychologique et sociale des personnes tout au long de la recherche et à assurer la confidentialité des informations recueillies. Je m'engage également à fournir aux participant.e.s tout le soutien permettant d'atténuer les effets négatifs pouvant découler de la participation à cette recherche. Pour ce faire, il est possible de me contacter par mail. *Liberté des participant.e.s :* le consentement pour poursuivre la recherche peut être retiré à tout moment sans donner de raison et sans encourir aucune responsabilité ni conséquence. Les réponses aux questions ont un caractère facultatif et le défaut de réponse n'aura aucune conséquence pour le sujet. *Information des participant.e.s :* les participant.e.s ont la possibilité d'obtenir des informations supplémentaires concernant cette étude auprès de l'investigateur, et ce dans les limites des contraintes du plan de recherche. Pour cela, merci de solliciter par mail Aurélie GONCALVES à l'adresse suivante : aurelie.goncalves@unimes.fr *Confidentialité des informations :* toutes les informations concernant les participant.e.s seront conservées de façon anonyme et confidentielle. Le traitement informatique n'est pas nominatif, il n'entre pas de ce fait dans la loi Informatique et Liberté. Cette recherche n'ayant qu'un caractère psychologique, elle n'entre pas de ce fait dans la loi Huriet-Sérusclat concernant la protection des personnes dans la recherche bio-médicale. La transmission des informations concernant le participant pour l'expertise ou pour la publication scientifique sera elle aussi anonyme. *Déontologie et éthique :* le promoteur et l'investigatrice s'engagent à préserver absolument la confidentialité et le secret professionnel pour toutes les informations concernant les participant.e.s (titre I, articles 1,3,5 et 6 et titre II, articles3, 9 et 20 du code de déontologie des psychologues, France).

Consentement

Je déclare accepter, librement, et de façon éclairée, de participer à cette étude

- ☐ J'accepte (1)
- ☐ Je refuse (2)

Début de bloc: Questions socio-démographiques

Quel âge avez-vous ? (écrivez juste le nombre)

Quel est votre sexe ?

- ☐ Un homme (1)
- ☐ Une femme (2)
- ☐ Autre (3)

Dans quelle.s filière.s êtes vous inscrit.e ? (si vous êtes en double cursus, indiquez vos filières dans autre)

- ☐ Psychologie (4)
- ☐ Droit (7)
- ☐ AES (5)
- ☐ Design (9)
- ☐ Sciences de la vie (6)
- ☐ STAPS (10)
- ☐ LLCER (11)
- ☐ Lettres (12)
- ☐ Mathématiques (13)
- ☐ Autre (14) ____

En quelle année d'étude êtes vous inscrit.e ? (si vous êtes en double cursus, indiquez l'année de la formation la plus avancée).

- ☐ L1 (2)
- ☐ L2 (8)
- ☐ L3 (3)
- ☐ M1 (4)
- ☐ M2 (5)
- ☐ Doctorat (6)
- ☐ Autre (7) ____

Veuillez indiquer à quel point, sur une échelle allant de 0 pour "pas du tout" à 100 pour "énormément", vous avez l'impression que

0	10	20	30	40	50	60	70	80	90	100

vos études universitaires sont essentielles pour vous ? ()	
la situation sanitaire vous pénalise dans votre projet professionnel ? ()	

Actuellement, dans quel type d'habitation logez-vous ?

- ☐ Logement personnel (1)
- ☐ Logement parental (2)

Quelle est la surface habitable (en m²) du logement dans lequel vous habitez ? (écrivez juste le nombre)

Actuellement, avec combien d'autres personnes êtes-vous dans ce logement ? (écrivez juste le nombre, si vous vivez seul.e, veuillez noter 0)

Avez vous changé de logement suite à la crise sanitaire?

- ☐ Oui (1)
- ☐ Non (2)

Précédemment, dans quel type d'habitation logiez-vous ?

- ☐ Logement personnel (1)
- ☐ Logement familial (4)

Etes-vous vacciné.e contre la COVID (ou en cours de schéma vaccinal)

- ☐ Oui (1)
- ☐ Non (4)

Depuis le début de la pandémie, avez-vous été diagnostiqué.e comme porteur du Covid 19 ?

- ☐ Oui (1)
- ☐ Non (2)

Depuis le début de la pandémie, combien de vos proches ont été diagnostiqués comme porteur du Covid-19 ? (écrivez juste le nombre, si aucun écrivez 0)

Actuellement, à quel point diriez vous que vous êtes inquiet.e (0 pas du tout - 100 complètement)

	0 10 20 30 40 50 60 70 80 90 100
Pour votre santé ()	
Pour la santé de vos proches ()	

Dans la série de questions ci-dessous,cochez la réponse qui exprime le mieux ce que vous avez éprouvé **au cours de la semaine qui vient de s'écouler**.

Je me sens tendu.e ou énervé.e

- ☐ Jamais (0)
- ☐ De temps en temps (1)
- ☐ Souvent (2)
- ☐ La plupart du temps (4)

Je prends plaisir aux mêmes choses qu'autrefois

- ☐ Oui, tout autant (0)
- ☐ Pas autant (1)
- ☐ Un peu seulement (2)
- ☐ Presque plus (3)

J'ai une sensation de peur comme si quelque chose d'horrible allait m'arriver

- ☐ Pas du tout (0)
- ☐ Un peu, mais cela ne m'inquiète pas (1)
- ☐ Oui, mais ce n'est pas trop grave (2)
- ☐ Oui, très nettement (3)

Je ris facilement et vois le bon côté des choses

- ☐ Autant que par le passé (0)
- ☐ Plus autant qu'avant (1)
- ☐ Vraiment moins qu'avant (2)
- ☐ Plus du tout (3)

Je me fais du souci

- ☐ Très occasionnellement (0)
- ☐ Occasionnellement (1)
- ☐ Assez souvent (2)
- ☐ Très souvent (3)

Je suis de bonne humeur

- ☐ La plupart du temps (0)
- ☐ Assez souvent (1)
- ☐ Rarement (2)
- ☐ Jamais (3)

Je peux rester tranquillement assis.e à ne rien faire et me sentir décontracté.e

- ☐ Oui, quoi qu'il arrive (0)
- ☐ Oui, en général (1)
- ☐ Rarement (2)
- ☐ Jamais (3)

J'ai l'impression de fonctionner au ralenti

- ☐ Jamais (0)
- ☐ Parfois (1)
- ☐ Très souvent (2)
- ☐ Presque toujours (3)

J'éprouve des sensations de peur et j'ai l'estomac noué

- ☐ Jamais (0)
- ☐ Parfois (1)
- ☐ Assez souvent (2)
- ☐ Très souvent (3)

Je ne m'intéresse plus à mon apparence

- ☐ J'y prête autant d'attention que par le passé (0)
- ☐ Il se peut que je n'y fasse plus autant attention (1)
- ☐ Je n'y accorde pas autant d'attention que je le devrais (2)
- ☐ Plus du tout (3)

J'ai la bougeotte et n'arrive pas à tenir en place

- ☐ Pas du tout (0)
- ☐ Pas tellement (1)
- ☐ Un peu (2)
- ☐ Oui, c'est tout à fait le cas (3)

Je me réjouis d'avance à l'idée de faire certaines choses

- ☐ Autant qu'avant (0)
- ☐ Un peu moins qu'avant (1)
- ☐ Bien moins qu'avant (2)
- ☐ Presque jamais (3)

J'éprouve des sensations soudaines de panique

- ☐ Jamais (0)
- ☐ Pas très souvent (1)
- ☐ Assez souvent (2)
- ☐ Vraiment très souvent (3)

Je peux prendre plaisir à un bon livre ou à une bonne émission de radio ou de télévision

- ☐ Souvent (0)
- ☐ Parfois (1)
- ☐ Rarement (2)
- ☐ Très rarement (3)

Nous vous demandons de répondre le plus honnêtement possible au questionnaire suivant. Il s'agit d'évaluer votre niveau d'accord ou de désaccord avec chacun des énoncés. Il n'y a pas de bonnes ou de mauvaises réponses.

	**1** Tout à fait d'accord (1)	**2** (2)	**3** (3)	**4** Ni d'accord, ni pas d'accord (4)	**5** (5)	**6** (6)	**7** Totalement en désaccord (7)
1. Il n'est pas possible que je puisse résoudre certains de mes problèmes (1)							
2. Parfois j'ai l'impression que je ne suis pas maitre de ma vie (2)							
3. J'ai peu de contrôle sur les choses qui m'arrivent (3)							
4. Je suis en capacité de faire à peu près tout ce que je souhaite (4)							
5. Je me sens souvent impuissant pour faire face aux difficultés que je rencontre dans la vie (5)							
6. Ce qui m'arrivera dans le futur dépend principalement de moi (6)							
7. Il y a peu de choses que je puisse faire pour changer les choses importantes de ma vie (7)							

Nous vous demandons de répondre le plus honnêtement possible au questionnaire suivant. Il s'agit d'évaluer votre niveau d'accord ou de désaccord avec chacun des énoncés. Il n'y a pas de bonnes ou de mauvaises réponses. **Lorsque vous y répondrez, essayez de penser aux personnes qui vous entourent**

	Fortement en désaccord (1)	En désaccord (2)	D'accord (3)	Fortement en accord (4)
Il y a des personnes sur qui je peux compter pour m'aider en cas de réel besoin (1)				
Il y a des personnes qui prennent plaisir aux mêmes activités sociales que moi (2)				
J'ai l'impression de faire partie d'un groupe de personnes qui partagent mes attitudes et mes croyances (3)				
J'ai des personnes proches de moi qui me procurent un sentiment de sécurité affective et de bien-être (4)				
Il y a quelqu'un avec qui je pourrais discuter de décisions importantes qui concernent ma vie (5)				
J'ai des relations où sont reconnus ma compétence et mon savoir-faire (6)				
Il y a une personne fiable à qui je pourrais faire appel pour me conseiller si j'avais des problèmes (7)				
Je ressens un lien affectif fort avec au moins une autre personne (8)				
Il y a des gens qui admirent mes talents et habiletés (9)				
Il y a des gens sur qui je peux compter en cas d'urgence (10)				

Fin de bloc: Provisions sociales

Début de bloc: MFI-20 Questionnaire de FATIGUE

Au moyen des énoncés suivants, nous souhaiterions estimer votre fatigue depuis 24 heures.
Par exemple pour la phrase "je me sens reposé.e", si vous pensez que cette phrase est entièrement vraie, que vous vous sentiez reposé.e récemment, veuillez cocher la case extrême gauche "Oui c'est vrai".
Si vous n'êtes pas entièrement d'accord avec cette phrase, selon le degré de désaccord, veuillez cocher l'une des possibilités dans la direction de "Non ce n'est pas vrai".

	**1** Oui c'est vrai (1)	**2** (2)	**3** (3)	**4** (4)	**5** Non ce n'est pas vrai (5)
Je me sens en forme (1)					
Physiquement, je ne me sens capable que de très peu (2)					
Je me sens très actif/active (3)					
J’ai envie de faire plein de choses agréables (4)					
Je me sens fatigué.e (5)					
Je pense que je fais beaucoup de chose dans une journée (6)					
Quand je fais quelque chose, je peux me concentrer dessus (7)					
Physiquement, je peux faire beaucoup (8)					
Je redoute d'avoir des choses à faire (9)					
Je pense que je ne fais pas grand-chose dans une journée (10)					
J'arrive à bien me concentrer (12)					
Je me sens reposé.e (13)					
Me concentrer sur quelque chose me demande beaucoup d'effort (14)					
Physiquement je me sens en mauvais état (15)					
J'ai un tas de projets (16)					
Je me fatigue facilement (17)					
Je mène peu de chose à bien (18)					
Je n'ai rien envie de faire (19)					
Mes pensées s'égarent facilement (20)					
Physiquement, je me sens en parfait état (21)					

Veuillez indiquer votre degré d'accord pour les affirmations suivantes

	1. Désaccord (1)	2 (2)	3 (3)	4 (4)	5 (5)	6. Accord (6)
J'ai tendance à me laisser influencer par les autres quand ils ont de fortes opinions (3)						
Je fais confiance à mes opinions, même quand elles vont à l'encontre du consensus (11)						
Je me juge par rapport à ce que je pense important et non pas selon les valeurs que les autres pensent importantes (13)						
En général, je me sens responsable des situations dans lesquelles je vis. (16)						
Souvent, les exigences de la vie quotidienne m'abattent (12)						
Je suis tout à fait valable dans la gestion des multiples responsabilités de ma vie quotidienne (6)						
Je pense qu'il est important d'avoir de nouvelles expériences qui mettent en cause la manière de penser aussi bien soi que le monde. (7)						
Pour moi, la vie est un processus continuel d'apprentissage, de changement et de croissance personnelle. (8)						
Il y a longtemps que j'ai renoncé à tenter de faire de grandes améliorations ou de grands changements dans ma vie (17)						
Il m'a été difficile et frustrant de maintenir des relations proches (18)						
Les gens me décriraient comme une personne qui donne et qui a la volonté de partager mon temps avec les autres (19)						
Je n'ai pas vécu beaucoup de relations chaleureuses et confiantes avec les autres (20)						
Je vis au jour le jour et ne pense pas vraiment au futur (21)						
Certaines personnes errent sans buts dans la vie : ce n'est pas mon cas (22)						
Souvent, je pense que j'ai fait tout ce qu'il y avait à faire dans la vie (23)						
Quand je regarde l'histoire de ma vie, je suis content.e de la manière dont les choses ont tourné. (24)						
J'apprécie la plupart des aspects de ma personnalité (25)						
À bien des égards, je me sens désappointé.e en ce qui concerne ce que j'ai réalisé dans ma vie. (26)						

Au cours du mois dernier, quand êtes-vous habituellement allé vous coucher le soir ? (exemple 22h30)____

Au cours du mois dernier, combien vous a-t-il habituellement fallu de temps (en minutes) pour vous endormir chaque soir ? (exemple : 60)____

Au cours du mois dernier, quand vous êtes-vous habituellement levé le matin ? (exemple : 7h30)____

Au cours du mois dernier, combien d'heures de sommeil effectif avez-vous eu chaque nuit ? (exemple 8h)____

Au cours du mois dernier, avec quelle fréquence avez-vous eu des troubles du sommeil car …

	Pas au cours du dernier mois (0)	Moins d'une fois par semaine (1)	Une ou deux fois par semaine (2)	Trois ou quatre fois par semaine (3)
vous n’avez pas pu vous endormir en moins de 30 minutes (4)				
vous vous êtes réveillé.e au milieu de la nuit ou précocement le matin (9)				
vous avez dû vous lever pour aller aux toilettes (6)				
vous n’avez pas pu respirer correctement (10)				
vous avez toussé ou ronflé bruyamment (11)				
vous avez eu trop froid (8)				
vous avez eu trop chaud (7)				
vous avez eu de mauvais rêves (5)				
vous avez eu des douleurs (12)				

Avez-vous eu des troubles du sommeil pour d'autre.s raison.s ? Si oui, décrivez brièvement ces raisons.

- ○ Non (5)
- ○ Oui (4) ____

Au cours du dernier mois...

	Pas au cours du dernier mois (1)	Moins d'une fois par semaine (5)	Une ou deux fois par semaine (3)	Trois ou quatre fois par semaine (4)
Indiquez la fréquence des troubles du sommeil pour ces raisons (4)	○	○	○	○

Au cours du mois dernier, comment évalueriez-vous globalement la qualité de votre sommeil ?

- ☐ Très bonne (0)
- ☐ Assez bonne (1)
- ☐ Assez mauvaise (2)
- ☐ Très mauvaise (3)

Au cours du mois dernier, combien de fois avez-vous pris des médicaments (prescrits par votre médecin ou achetés sans ordonnance) pour faciliter votre sommeil ?

- ☐ Pas au cours du dernier mois (0)
- ☐ Moins d'une fois par semaine (1)
- ☐ Une ou deux fois par semaine (2)
- ☐ Trois ou quatre fois par semaine (3)

Au cours du mois dernier, combien de fois avez-vous eu des difficultés à demeurer éveillé.e pendant que vous conduisiez, preniez vos repas, étiez occupé.e dans une activité sociale ?

- ☐ Pas au cours du dernier mois (0)
- ☐ Moins d'une fois par semaine (1)
- ☐ Une ou deux fois par semaine (2)
- ☐ Trois ou quatre fois par semaine (3)

Au cours du mois dernier, à quel degré cela a-t-il représenté un problème pour vous d'avoir assez d'enthousiasme pour faire ce que vous aviez à faire ?

- ☐ Pas du tout un problème (0)
- ☐ Seulement un tout petit problème (1)
- ☐ Un certain problème (2)
- ☐ Un très gros problème (3)

Pour chacune des caractéristiques ou descriptions suivantes, cochez la case qui vous décrit le mieux

	**1** Jamais (1)	**2** (2)	**3** (3)	**4** (4)	**5** Toujours (5)
1. Je respecte mon corps. (1)					
2. Je me sens bien à propos de mon corps. (2)					
3. Je sens que mon corps présente au moins certaines bonnes qualités. (3)					
4. Je prends une attitude positive envers mon corps. (4)					
5. Je suis attentif/ve aux besoins de mon corps. (5)					
6. Je ressens de l'amour pour mon corps. (7)					
7. J'apprécie les caractéristiques différentes et uniques de mon corps. (8)					
8. Mes comportements révèlent mon attitude positive envers mon corps (ex: je garde ma tête haute et je souris.) (9)					
9. Je suis confortable dans mon corps. (10)					
10. Je sens que je suis beau/belle même si je suis différent/e des images de beautés véhiculées dans les médias (ex: mannequins, actrices/acteurs). (27)					

Pour chacune des caractéristiques ou descriptions suivantes, cochez la case qui vous décrit le mieux

	1<br>Jamais (0)	2<br>Rarement (0)	3<br>Quelque fois (0)	4<br>Souvent (1)	5<br>Très souvent (2)	6<br>Toujours (2)
1. Je suis terrifié.e à l'idée d'être trop gros.se. (1)						
2. J'évite de manger quand j'ai faim (2)						
3. Je suis trop soucieux.se de la nourriture. (3)						
4. J'ai fait des excès alimentaires au cours desquels je pensais ne pas pouvoir m'arrêter. (4)						
5. Je coupe ma nourriture en petits morceaux (5)						
6. J'ai conscience de la valeur calorique des aliments que je mange. (6)						
7. J'évite particulièrement les aliments tels le sucre, le pain ou les pommes de terre (7)						
8. J'ai l'impression que les autres préféreraient que je mange davantage. (8)						
9. Je vomis après avoir mangé. (9)						
10. Je me sens très coupable après avoir mangé. (10)						
11. Le désir d'être plus mince me préoccupe. (11)						
12. Quand je me dépense physiquement il me vient à l'idée que je brule des calories. (12)						
13. Les autres pensent que je suis trop mince. (13)						
14. Je suis préoccupé.e par le fait d'avoir trop de graisse dans le corps. (14)						
15. Je prends plus de temps que les autres à prendre mes repas. (15)						
16. J'évite de manger des aliments trop sucrés. (16)						
17. Je mange des aliments diététiques. (17)						
18. J'ai l'impression que la nourriture domine ma vie. (18)						
19. Je parle volontiers de mes capacités à contrôler mon alimentation. (19)						
20. J'ai l'impression que les autres me poussent à manger (20)						
21. Je consacre trop de temps et pense trop à la nourriture. (21)						
22. Je me sens mal à l'aise après avoir mangé des sucreries (22)						
23. Je m'oblige à me mettre à la diète (23)						

24. J'aime que mon estomac soit vide. (24)

25. Je déteste essayer de la nouvelle nourriture riche en calories. (25)

26. J'ai envie de vomir après les repas. (26)

Considérez-vous que vous êtes un minimum actif/active au quotidien ? (ex : marche, sortir votre chien, jardiner, faire le ménage...)

○ Oui (1)

○ Non (2)

Indiquez dans quelle mesure chacun des énoncés suivants correspond actuellement à l'une des raisons pour lesquelles vous pratiquez des activités physiques

	Ne correspond pas du tout (1)	Correspond très peu (2)	Correspond un peu (3)	Correspond moyennement (4)	Correspond assez (5)	Correspond fortement (6)	Correspond très fortement (7)
1. Pour le plaisir que je ressens lorsque je pratique des activités physiques (1)							
2. Je n'en ai aucune idée, je crois que ça ne me sert à rien (2)							
3. Parce que je me sentirais mal si je ne faisais pas cet effort (3)							
4. Parce que je pense que l'activité physique est une bonne chose pour mon développement personnel (4)							
5. Je ne sais pas vraiment; j'ai l'impression de perdre mon temps lorsque je fais de l'activité physique (5)							
6. Pour la satisfaction que je ressens à progresser dans ce genre d'activités (6)							
7. Parce que l'activité physique fait partie intégrante du style de vie que j'ai choisi (7)							
8. Franchement je fais de l'activité physique mais je ne vois pas l'intérêt (8)							
9. Pour ne pas avoir à entendre les reproches de certaines personnes (9)							
10. Parce que je considère que faire de l'activité physique est une partie de mon identité (10)							
11. Pour les sensations agréables que me procure l'activité physique (11)							
12. Parce que personnellement je considère que c'est un facteur de bien-être (12)							
13. Parce que faire de l'activité physique est cohérent avec mes valeurs (13)							

14. Parce que je me sentirais nerveux-se si je n'en faisais pas (14)
15. Parce que certaines personnes me mettent la pression pour que je le fasse (15)
16. Parce que je pense que l'activité physique me permettra de me sentir mieux... (16)
17. Parce que j'y suis obligé(e) par mon entourage (17)
18. Parce que je dois le faire pour me sentir bien avec moi-même. (18)

Nous souhaiterions en savoir un peu plus sur votre niveau actuel d'activité physique. Nous vous remercions de bien lire les questions et d'y répondre le plus juste possible.

1-a. Au cours des **7 derniers jours**, combien y a-t-il eu de jours au cours desquels vous avez fait des **activités physiques intenses** comme porter des charges lourdes, bêcher, faire du VTT ou jouer au football ?

○ Nombre de jours (1) ____

○ Je n'ai pas fait d'activité physique intense au cours des 7 derniers jours (0)

1-b. Au total, combien de **temps** avez-vous passé à faire des activités intenses **au cours des 7 derniers jours ?**
Ex : si 1h45 --> case nombre d'heure = 1 ; case nombre de minutes = 45

- ☐ Nombre d'heures (1) ____
- ☐ Nombre de minutes (2) ____
- ☐ Je ne sais pas (0)

2. Pensez à toutes les **activités modérées** que vous avez faites au cours **des 7 derniers jours.**
Les activités physiques modérées font référence aux activités qui vous demandent un effort physique modéré et vous font respirer un peu plus difficilement que normalement. Pensez seulement aux activités que vous avez effectuées pendant **au moins 10 minutes d'affilée.**

2-a. Au cours des **7 derniers jours**, combien y a-t-il eu de jours au cours desquels vous avez fait des **activités physiques modérées** comme porter des charges légères, passer l'aspirateur, faire du vélo tranquillement ou jouer au volley-ball ? **Ne pas inclure la marche.**

◯ Nombre de jours (chiffre compris entre 1 et 7) (1)
____

◯ Je n'ai pas eu d'activité physique modérée (0)

2-b. Au total, combien de temps avez-vous passé à faire des activités modérées au cours des 7 derniers jours ?

Ex : si 1h45 --> case nombre d'heures = 1 ; case nombre de minutes = 45

- ☐ Nombre d'heures (1) ____
- ☐ Nombre de minutes (2) ____
- ☐ Je ne sais pas (0)

3. Pensez au temps que vous avez passé à marcher **au moins 10 minutes d'affilée** au cours des **7 derniers jours.**
Cela comprend la marche à l'université et à la maison, la marche pour vous rendre d'un lieu à un autre, et tout autre type de marche que vous auriez pu faire pendant votre temps libre pour la détente, le sport ou les loisirs.

3-a. Au cours des 7 derniers jours, combien y a-t-il eu de jours au cours desquels vous avez marché pendant au moins 10 minutes d'affilée.

◯ (nombre de jours) (1) ____

◯ Je n'ai pas fait de marche (0)

3.b. Au total, combien d'épisodes de marche d'au **moins 10 minutes d'affilée** avez-vous

effectué au cours des **7 derniers jours** ? (vous trouverez ci-dessous un exemple sur une semaine)

○ nombre d'épisodes de 10 minutes d'affilée (1) ____

○ Je ne sais pas (0)

4. La dernière question porte sur le temps **que vous avez passé assis** pendant les jours de semaine, au cours des **7 derniers jours.** Cela comprend le temps passé assis à l'université, à la maison, lorsque vous étudiez et pendant votre temps libre. Il peut s'agir par exemple du temps passé assis à un bureau, chez des amis, à lire, à être assis ou allongé pour regarder la télévision, devant un écran.

4-a. Au cours des 7 derniers jours, pendant les jours de semaine, combien de temps, **en moyenne par jour,** avez vous passé assis ? (p.ex., si vous êtes resté.e assis.e 6h le lundi, 5h le mardi, 5h le mercredi, 4h le jeudi et 5h le vendredi, indiquez 5 à nombre d'heures)

- ☐ nombre d'heures (1) ____
- ☐ nombre de minutes (2) ____
- ☐ Je ne sais pas (0)

Nous vous remercions de votre investissement dans cette étude et de votre confiance. Merci d'indiquer l'adresse mail à laquelle nous pourrons vous envoyer le questionnaire suivant (votre adresse mail sera séparée de vos réponses pour garantir l'anonymat)

____

Afin de garantir l'anonymat de vos réponse merci d'indiquer un code en suivant les règles suivantes :
- Les trois premières lettres de votre prénom
- Les deux derniers chiffres de votre année de naissance

- Les deux premières lettres de votre nom

Par exemple Marie Dupond, née en 1980 a le code : MAR80DU
