# Supplementary material for: Promoting Physical Activity Among University Students During the COVID-19 Pandemic: Protocol for a Randomized Controlled Trial
Source: JMIR Res Protoc. 2022 Jun 13;11(6):e36429. doi: 10.2196/36429 (PMC9202516; doi:10.2196/36429)
Supplement: Multimedia Appendix 2 [file resprot_v11i6e36429_app2.pdf]

	Résilience - COVID-19	
		**Edition 2021**

**Projet / Proposal**	
**Acronyme / Acronym**	COV'Etu
**Titre / Title**	Conséquences de la COVID-19 sur la santé des étudiants : effets sur leur mode de vie et leur état psychologique

**Coordinateur du projet**			
**Prénom / First Name**	Elodie	**Nom / Last Name**	CHARBONNIER
**Téléphone / Phone**		**Email**	elodie.charbonnier@unimes.fr
**Organisme / Institution**	APSY-v		

RETOUR AU COORDINATEUR / COMMITTEE REPORT

1. Décision du comité / Decision of the committee

Ce projet vise à appréhender les conséquences à long terme de la COVID-19 auprès des étudiants. Il s'organise en 2 axes : le premier est évaluatif et le second plus interventionnel. Dans le premier volet, il s'agit d'appréhender l'état psychologique et divers déterminants plutôt individuels mais aussi situationnels, psychosociaux. Le second volet évalue les effets sur la santé d'un programme axé sur le stress et l'apprentissage et un autre sur l'activité physique via un groupe expérimental et un groupe contrôle et la co-construction avec les étudiants. Le design de l'étude est solide, le recours aux diverses méthodologies est clair et bien explicité. Les retombées scientifiques et sociétales sont importantes et très bien présentées. L'équipe a travaillé sur la COVID-19 depuis un an et déjà valorisé ses travaux Ce projet est très solide, très bien décrit, il a un caractère d'urgence évident. Le comité d'évaluation l'a ainsi considéré comme prioritaire pour financement.

Projet	
**Acronyme**	**COV'Etu**
**Titre en français**	**Conséquences de la COVID-19 sur la santé des étudiants : effets sur leur mode de vie et leur état psychologique**
**Titre en anglais**	**Health consequences of COVID-19 on university students: effects on the lifestyle and psychological health**
**Instrument financier**	**Autres actions spécifiques ponctuelles**

Coordinateur du projet			
**Prénom**	Elodie	**Nom**	CHARBONNIER
**Email**	elodie.charbonnier@unimes.fr		
**Organisme**	APSY-v		

Caractère d'urgence	
Caractère d'urgence, ciblant l'acquisition de connaissances avec une application attendue des résultats, méthodes ou techniques dans les 3 à 12 mois suivant l'attribution du financement, ou projet justifiant un recueil immédiat de données spécifiques à la période épidémique et post-épidémique.<br><br>*Ce critère revêt un caractère discriminant.*<br><br>*Donner une note de 0 à 5 (indiquer les forces et les faiblesses sous forme de liste)*	
Ce projet de recherche aborde des thématiques d'une urgence criante dans le contexte sanitaire actuel. Il permettra d'obtenir des connaissances précieuses de l'état psychosocial des étudiants universitaires et des leviers possibles afin d'augmenter leur bien-être psychologique. Je soutiens fortement l'initiative des chercheurs impliqués dans ce projet.	

**Qualité des objectifs du projet et de sa méthodologie au regard des thématiques ciblées par l'appel à projets.**	
Thématiques visées par l'appel:<br>*Physiopathogénie et épidémiologie :*<br>*- Immunité mucosale ;*<br>*- Nouveaux modèles de recherche préclinique afin de valider des approches thérapeutiques innovantes*<br>*- Conséquences à moyen et à long terme de la Covid-19 chez les patients infectés (pathologies post Covid-19 et révélation de pathologies sous-jacentes par la Covid-19) ;*<br>*- Impact de la pandémie sur les patients souffrant de pathologies non-Covid-19 et leur prise en charge ;*<br>*Prévention, contrôle de l'épidémie et dynamiques sociales :*<br>*- Organisation du système de santé, de soin et de prévention face à la pandémie Covid-19 : rôles des différents acteurs publics et privés, comparaison internationale ;*<br>*- Conséquences à court et long terme de la Covid-19 sur la santé mentale et le bien être psychologique dans les différents groupes de population ;*<br>*- Analyse et évolution des politiques de lutte, du rôle des différents acteurs publics ou privés et des différents secteurs d'activité ; comparaisons interrégionales et internationales, production des normes et des pratiques ;*<br>*- Effet de l'épidémie sur les inégalités, la vulnérabilité et la précarité des populations ;*<br>*- Evolution des formes de sociabilité, de cohésion sociale et des comportements collectifs ;*<br>*Impacts économique et organisationnel sur les différents secteurs d'activité et les relations internationales*<br><br>*Donner une note de 0 à 5 (indiquer les forces et les faiblesses sous forme de liste)*	
Le projet de recherche me parait bien construit. La présence de deux axes (diagnostique et interventionnel) parait, selon moi, constituer une importante plus-value.<br><br>La prise en compte de facteurs « explicatifs » aux niveau individuel et situationnel au niveau du premier axe, constitue également un aspect positif du projet. Le fait que la collecte de données ait été précédée par une collecte antérieure de type longitudinal (en 2020), est également intéressant.<br><br>J'aurais apprécié lire une description des méthodes statistiques envisagées afin de mettre en évidence les rôles spécifiques joués par les différents facteurs individuels et situationnels (régressions hiérarchiques, analyses multiniveaux ?).<br><br>Une question que je me pose concernant la première collecte de données, concerne son caractère intra ou inter-sujets. J'imagine que les auteurs essayeront d'apparier un maximum d'observations (via, par exemple, un code demandé aux quatre temps de mesure) mais cela ne m'est pas apparu clairement.<br><br>L'utilisation d'entretiens dans le second axe me parait constituer une plus-value intéressante. Ne serait-il pas envisageable de réaliser également une série d'entretiens dans le cadre du premier axe, afin d'illustrer les résultats	

quantitatifs ?<br><br>De manière générale, les objectifs et la méthodologie proposés sont, selon moi, de grande qualité et d'une importance majeure dans le contexte actuel.	

**Qualité et compétences du consortium ou de l'équipe permettant d'atteindre les objectifs visés par le projet.**	
Donner une note de 0 à 5 (indiquer les forces et les faiblesses sous forme de liste)	
Les membres de l'équipe me paraissent tout à fait à même de réaliser le projet de recherche proposé. Le caractère multidisciplinaire des profils et des méthodologies de recherche constitue selon moi, une plus-value décisive.	

**Conclusion de l'évaluation**	

Projet	
**Acronyme**	**COV'Etu**
**Titre en français**	**Conséquences de la COVID-19 sur la santé des étudiants : effets sur leur mode de vie et leur état psychologique**
**Titre en anglais**	**Health consequences of COVID-19 on university students: effects on the lifestyle and psychological health**
**Instrument financier**	**Autres actions spécifiques ponctuelles**

Coordinateur du projet			
**Prénom**	Elodie	**Nom**	CHARBONNIER
**Email**	elodie.charbonnier@unimes.fr		
**Organisme**	APSY-v		

Caractère d'urgence

Caractère d'urgence, ciblant l'acquisition de connaissances avec une application attendue des résultats, méthodes ou techniques dans les 3 à 12 mois suivant l'attribution du financement, ou projet justifiant un recueil immédiat de données spécifiques à la période épidémique et post-épidémique.

Ce critère revêt un caractère discriminant.

Donner une note de 0 à 5 (indiquer les forces et les faiblesses sous forme de liste)

Le projet expertisé est très important dans la mesure où il s'attache à la fois à déterminer les problèmes rencontrés par les étudiants du fait de la pandémie. Il se veut être une aide à améliorer le soutien aux étudiants pour leurs études mais également pour conforter leur santé psychologique ainsi que leur mode de vie, ceci après avoir identifié les facteurs individuels et situationnels susceptibles d'avoir des conséquences délétères pour les étudiants. Étant donné le mal-être de nombre de ces jeunes repérés depuis un an, il est de fait urgent de les aider à améliorer substantiellement leur santé et leur bien-être.

**Qualité des objectifs du projet et de sa méthodologie au regard des thématiques ciblées par l'appel à projets.**	
Thématiques visées par l'appel:<br>*Physiopathogénie et épidémiologie :*<br>*- Immunité mucosale ;*<br>*- Nouveaux modèles de recherche préclinique afin de valider des approches thérapeutiques innovantes*<br>*- Conséquences à moyen et à long terme de la Covid-19 chez les patients infectés (pathologies post Covid-19 et révélation de pathologies sous-jacentes par la Covid-19) ;*<br>*- Impact de la pandémie sur les patients souffrant de pathologies non-Covid-19 et leur prise en charge ;*<br>*Prévention, contrôle de l'épidémie et dynamiques sociales :*<br>*- Organisation du système de santé, de soin et de prévention face à la pandémie Covid-19 : rôles des différents acteurs publics et privés, comparaison internationale ;*<br>*- Conséquences à court et long terme de la Covid-19 sur la santé mentale et le bien être psychologique dans les différents groupes de population ;*<br>*- Analyse et évolution des politiques de lutte, du rôle des différents acteurs publics ou privés et des différents secteurs d'activité ; comparaisons interrégionales et internationales, production des normes et des pratiques ;*<br>*- Effet de l'épidémie sur les inégalités, la vulnérabilité et la précarité des populations ;*<br>*- Evolution des formes de sociabilité, de cohésion sociale et des comportements collectifs ;*<br>*Impacts économique et organisationnel sur les différents secteurs d'activité et les relations internationales*<br><br>*Donner une note de 0 à 5 (indiquer les forces et les faiblesses sous forme de liste)*	
Le projet s'inscrit dans la thématique "Conséquences à court et long terme de la Covid-19 sur la santé mentale et le bien-être psychologique" ici dans une population étudiante.<br>Il se compose de deux axes de recherche, le premier s'attelle à définir les facteurs sur lesquels il est nécessaire d'agir pour éviter davantage de dégradation de la santé psychologique et du mode de vie perturbateur des étudiants universitaires.<br>Le deuxième axe évalue les effets sur la santé d'un programme axé sur le stress et l'apprentissage et un autre sur l'activité physique, ceci avec un objectif très intéressant, à savoir apporter des résultats permettant à toutes les universités de prendre de bonnes décisions pour réduire l'impact de la pandémie sur les étudiants. L'équipe a prévu de mettre en place des situations de prévention.<br>Un autre point fort est l'approche pluridisciplinaire de ce projet, psychologie clinique, psychologie cognitive, innovation sociale par le design, nutrition et activité physique, seul moyen d'aider vraiment l'amélioration du bien-être des étudiants universitaires.<br>La population est constituée d'étudiants universitaires, soit de jeunes adultes provenant de milieux sociaux très différents, ce qui est une force de ce projet.<br>La littérature scientifique sur laquelle s'appuie le projet est tout à fait intéressante, car elle ne s'appuie pas que sur des articles récents liés à la pandémie, mais prend également en compte ce qu'on sait depuis longtemps sur le fait que les étudiants universitaires sont identifiés comme une population vulnérable.<br>Les explications données par l'équipe sur les connaissances que l'on a sur les effets de la pandémie sont tout à fait justifiées et très bien étayées.	

Les résultats obtenus par l'équipe sur des recherches effectuées en 2020, justifiant le projet présent, sont tout à fait convaincants.<br>2000 étudiants universitaires sont prévus d'être inclus dans ce projet, à l'identique de ce qui avait été fait dans leurs recherches précédentes.<br>Les variables étudiées sont tout à fait pertinentes dans le cadre des deux axes de recherche.<br>Dans le deuxième axe de recherche, interventionniste, est parfaitement bien prévu puisqu'il joue sur la co-construction avec les utilisateurs des programmes, en vue de les rendre acteurs des changements à venir.<br>Pour ce deuxième axe, deux groupes sont constitués, un expérimental et un contrôle, avec pour chacun de ces groupes deux mesures, à la même période, soit avant la mise en place du programme pour le groupe expérimental puis après la fin de ce programme.<br>Ces programmes contiennent des interventions innovantes pour réduire et / ou prévenir la détérioration de la santé mentale et des difficultés d'apprentissage, interventions dont l'efficacité a été prouvée.<br>Un aspect très intéressant de ce projet est que l'équipe a prévu de mettre à disposition de toutes les universités françaises leurs programmes, afin qu'elles puissent les répliquer.<br>Une promotion de l'information est prévue par le biais d'une conférence auprès des services de la médecine préventive.<br>L'équipe considère que ces programmes resteront pertinents pour les universités, même au-delà de la crise sanitaire.	

**Qualité et compétences du consortium ou de l'équipe permettant d'atteindre les objectifs visés par le projet.**	
Donner une note de 0 à 5 (indiquer les forces et les faiblesses sous forme de liste)	
Comme cela a été annoncé, l'équipe a une expertise pluridisciplinaire bienvenue, Psychologie clinique, Psychologie du sport et des sciences, Nutrition et Santé publique, Psychologie cognitive, Design, Psychologie de la santé, Psychologie clinique de la Santé, ce qui est le gage d'une recherche prenant en compte tous les facteurs susceptibles d'agir sur la santé et le bien-être des étudiants universitaires.<br>Les facteurs individuels et situationnels ne peuvent qu'être réellement pris en considération, ce qui est particulièrement important dans une telle étude.	

**Conclusion de l'évaluation**	
Mon avis sur ce projet est tout à fait positif, les deux axes de la recherche vont permettre à la fois de mettre en évidence les facteurs sur lesquels il est indispensable d'agir mais également d'apporter aux étudiants universitaires des aides qu'ils pourront utiliser y compris lorsque, espérons le, la pandémie sera enfin éloignée.<br>Je donne un avis très favorable à ce projet de recherche.	
